# Extracting Teeth

**Published:** 1867-08

**Authors:** M. S. Jackson

**Affiliations:** Oskaloosa, Iowa


### EXTRACTING TEETH.

BY DR. M. S. JACKSON, OSKALOOSA, IOWA.

DEAR REGISTER:—Permit me through your columns to give a few thoughts on the subject of extracting teeth. Although much has been said and written upon it, there yet remains much to be learned on this important branch of Dental practice. There has been much said of the policy of extracting teeth without the use of the lancet. Many objections have been urged against it, and many advantages brought forward to sustain it. One writer publishes it to the world as his peculiar style of extracting teeth; and another criticises his remarks, and calls his practice a hobby.

Yet none of these writers, from the venerable Dr. Harris down to Dr. —— of the present day, says one word about TIME in the operation. Do we ever see an account of a surgical operation, without seeing the length of time noted in which it took to perform that operation? Do we see or hear of any great bodily suffering, without instinctively asking or wondering how long such suffering lasted?

In childbirth, for instance, what a shudder it produces to hear that a delicate mother has suffered all night in labor! and how pleasant, on the other hand, to know that her suffering was all over in half an hour!

Dr. Harris says there are few cases in surgery that cause more dread than the extraction of teeth; and yet do we not work away at this painful operation with the most perfect indifference as it regards time?

And as time is the essence of many great contracts, so it should ever be in all operations for the extraction of teeth, always to get through with the operation as soon as possible, and never prolong it; not even at the suggestion of the patient, beyond the time actually necessary for the removal of the teeth, and you will be surprised (if you have never tried) how much time, and consequently suffering, you can save, and how many teeth you can extract in a few moments, and a majority of them, too, without the knowledge of the patient. My incredulous friend asks how this can be done without the aid of anæsthesia. It is simply this: It is a well known fact that a sharp hurt, such as a cut or bruise, deadens the sensibilities of the surrounding parts; and when one tooth has been extracted, if there be four or six adjoining, they can also be removed before reaction takes place, and the pain consequent upon the first extraction is about all that is experienced. I have seen first-class operators, in extracting a number of teeth, average one in about ten minutes; when they should have taken out ten in one minute. My mode for extracting teeth is this (and I give it not as my peculiar style of operating). I know not how many do the same; I know some, however, who do not:

When a patient calls to have a number of teeth removed, I examine them thoroughly to ascertain what advantages, if any, can be taken, and where the greatest number can be taken from without changing my forceps, always beginning with the easiest one of this selected number. And here, let me remark, that I seldom use the lancet, unless it be for fangs, or teeth where the gum is very firm and hard. My experience goes to prove (to me at least) that in nine cases out of ten where it is necessary to remove the teeth, the gums are soft and loose from them. And if there has been no anæsthetic given, I bathe the gum of the tooth selected with chloroform, and extract it and as many more as the flow of blood will permit, without letting go of the patient's head. In this way I have often taken out six, eight, and ten, and when the patient really thought I was all the while at one tooth, and, in a majority of cases, when questioned on the subject, will say, why you pulled them so quickly I didn't know it! I thought you were all this time taking out the first tooth! and, in many instances, they will go away and say you put them under the influence of chloroform and took their teeth out without their knowing that you had given them any thing.

I will give only two instances that occurred in my office recently, both patients being highly respectable ladies of this place:

Mrs. T——, aged forty-five, bilious temperament, applied to have her lower teeth taken out, eight in all, four incisors, two cuspids, and two bicuspids. I had her seated, and selected the right lateral incisor to begin with, bathed the gum with chloroform, and took it and the remaining seven teeth out without letting go of her head. When the last one was extracted, (it being the hardest one of all) I let go of her head, when she sprang to her feet and said I should pull no more teeth for her, as I had been all this time taking out one. I laughingly remarked that she could keep her remaining teeth, I should not trouble her further. Her husband, who had remained in the reception room, heard her talking excitedly, came in, and insisted upon her resuming her seat and have the others taken out. At this moment she put her finger to her mouth, to show her husband how I had treated her (saying her mouth felt so strangely), when she discovered her teeth were all gone. At this she gave utterance to some of the most ludicrous expressions of gratitude I ever heard; and insists that she knew of but one being taken out.

Mrs. P——, aged forty, of full habit, presented herself to have six upper teeth taken out, one cuspid, one bicuspid, and one molar on the right side; on the left were one bicuspid and two molars, all firmly set. I selected the bicuspid on the right side to begin with, bathed as before with chloroform, and took out the entire six in about fifteen seconds, and without the slightest motion, or expression of pain from her. And when told that her teeth were all out, she would not believe it, until she had felt repeatedly for them, and still insists that she never knew when any were taken out after the first one. That there are exceptions to these cases, I do not pretend to deny. Where the teeth are very hard to extract, or are broken off under the gums, such rapidity can not be practiced; but in these cases much time and suffering can be saved by the Dentist if he chooses to do so. And should it not be his first duty to his patients to save them from as much pain as possible, ever remembering that the longer a painful operation lasts the more exhaustion is produced, and the more dread the patient has to submit to a similar operation.

